# Advancements in Immunity and Dementia Research: Highlights from the 2023 AAIC Advancements: Immunity Conference

**DOI:** 10.1002/alz.14291

**Published:** 2024-12-18

**Authors:** Courtney M. Kloske, Simin Mahinrad, Christopher J. Barnum, Andre F. Batista, Elizabeth M. Bradshaw, Brittany Butts, Maria C. Carrillo, Paramita Chakrabarty, Xiaoying Chen, Suzanne Craft, Sandro Da Mesquita, Luke C. Dabin, Davangere Devanand, Violeta Duran‐Laforet, Wassim Elyaman, Elizabeth E. Evans, Patricia Fitzgerald‐Bocarsly, Kate E. Foley, Ashley S. Harms, Michael T. Heneka, Soyon Hong, Yu‐Wen A. Huang, Stephanie Jackvony, Laijun Lai, Yann Le Guen, Cynthia A. Lemere, Shane A. Liddelow, Alfonso Martin‐Peña, Anna G. Orr, Francisco J. Quintana, Grace D. Ramey, Jessica E. Rexach, Stacey J. S. Rizzo, Claire Sexton, Alice S. Tang, Jose G. Torrellas, Andy P. Tsai, Lynn van Olst, Keenan A. Walker, Whitney Wharton, Malú Gámez Tansey, Donna M Wilcock

**Affiliations:** ^1^ Alzheimer's Association Chicago Illinois USA; ^2^ INmune Bio, Inc. Boca Raton Florida USA; ^3^ Ann Romney Center for Neurologic Diseases Brigham and Women's Hospital Harvard Medical School Boston Massachusetts USA; ^4^ Department of Neurology The Carol and Gene Ludwig Center for Research on Neurodegeneration, Division of Translational Neurobiology Columbia University New York New York USA; ^5^ Emory University Atlanta Georgia USA; ^6^ Department of Neuroscience Center for Translational Research in Neurodegenerative Diseases University of Florida Gainesville Florida USA; ^7^ Department of Neurology, Hope Center for Neurological Disorders, Knight Alzheimer's Disease Research Center Washington University School of Medicine St. Louis Missouri USA; ^8^ Alzheimer's Disease Research Center Wake Forest University School of Medicine Winston‐Salem North Carolina USA; ^9^ Department of Neuroscience Mayo Clinic Jacksonville Florida USA; ^10^ Department of Medical & Molecular Genetics Indiana University School of Medicine Indianapolis Indiana USA; ^11^ Stark Neuroscience Research Institute Indiana University School of Medicine Indianapolis Indiana USA; ^12^ Columbia University Irving Medical Center New York New York USA; ^13^ Department of Neurobiology Brudnick Neuropsychiatric Research Institute University of Massachusetts Chan Medical School Worcester Massachusetts USA; ^14^ Department of Neurology Division of Translational Neurobiology Columbia University Irving Medical Center New York New York USA; ^15^ The Taub Institute for Research on Alzheimer's Disease and the Aging Brain Columbia University Irving Medical Center New York New York USA; ^16^ Vaccinex, Inc Rochester New York USA; ^17^ Rutgers New Jersey Medical School Newark New Jersey USA; ^18^ Department of Neurology Stark Neurosciences Research Institute Indiana University Indianapolis Indiana USA; ^19^ Department of Neurology University of Alabama at Birmingham Birmingham Alabama USA; ^20^ Luxembourg Centre for Systems Biomedicine University of Luxembourg Belval Luxembourg; ^21^ Division of Infectious Diseases and Immunology University of Massachusetts Medical School North Worcester Massachusetts USA; ^22^ UK Dementia Research Institute at University College London Institute of Neurology London UK; ^23^ Brown University Providence Rhode Island USA; ^24^ Appel Alzheimer's Disease Research Institute Feil Family Brain and Mind Research Institute Neuroscience Graduate Program Weill Cornell Medicine New York New York USA; ^25^ University of Connecticut Storrs Connecticut USA; ^26^ Department of Neurology and Neurological Sciences Stanford University Palo Alto California USA; ^27^ Department of Medicine Quantitative Sciences Unit Stanford University School of Medicine Palo Alto California USA; ^28^ Department of Neurology Ann Romney Center for Neurologic Diseases Brigham and Women's Hospital Boston Massachusetts USA; ^29^ Departments of Neurology Harvard Medical School Boston Massachusetts USA; ^30^ Departments of Neuroscience & Physiology and Ophthalmology Neuroscience Institute NYU Grossman School of Medicine New York New York USA; ^31^ Department of Neuroscience Center for Translational Research in Neurodegenerative Disease McKnight Brain Institute University of Florida Gainesville Florida USA; ^32^ Gene Lay Institute for Immunology and Inflammation Boston Massachusetts USA; ^33^ The Broad Institute of MIT and Harvard Boston Massachusetts USA; ^34^ Biological and Medical Informatics PhD Program University of California San Francisco San Francisco California USA; ^35^ Bakar Computational Health Sciences Institute University of California San Francisco San Francisco California USA; ^36^ Program in Neurogenetics Department of Neurology David Geffen School of Medicine University of California Los Angeles Los Angeles California USA; ^37^ Aging Institute University of Pittsburgh School of Medicine Pittsburgh Pennsylvania USA; ^38^ Graduate Program in Bioengineering University of California, San Francisco and University of California, Berkeley San Francisco California USA; ^39^ Center for Translational Research in Neurodegenerative Disease University of Florida Gainesville Florida USA; ^40^ Department of Neurology and Neurological Sciences Stanford University School of Medicine Stanford California USA; ^41^ Wu Tsai Neurosciences Institute Stanford University Stanford California USA; ^42^ The Ken & Ruth Davee Department of Neurology Northwestern University Feinberg School of Medicine Chicago Illinois USA; ^43^ Laboratory of Behavioral Neuroscience National Institute on Aging (NIA) Intramural Research Program Baltimore Maryland USA; ^44^ School of Nursing Emory University Atlanta Georgia USA; ^45^ Department of Neuroscience University of Florida College of Medicine McKnight Brain Institute Norman Fixel Institute for Neurological Diseases Gainesville Florida USA

**Keywords:** Alzheimer's disease, immunity, inflammation, microglia, therapeutics

## Abstract

The immune system is a key player in the onset and progression of neurodegenerative disorders. While brain resident immune cell‐mediated neuroinflammation and peripheral immune cell (eg, T cell) infiltration into the brain have been shown to significantly contribute to Alzheimer's disease (AD) pathology, the nature and extent of immune responses in the brain in the context of AD and related dementias (ADRD) remain unclear. Furthermore, the roles of the peripheral immune system in driving ADRD pathology remain incompletely elucidated. In March of 2023, the Alzheimer's Association convened the Alzheimer's Association International Conference (AAIC), Advancements: Immunity, to discuss the roles of the immune system in ADRD. A wide range of topics were discussed, such as animal models that replicate human pathology, immune‐related biomarkers and clinical trials, and lessons from other fields describing immune responses in neurodegeneration. This manuscript presents highlights from the conference and outlines avenues for future research on the roles of immunity in neurodegenerative disorders.

**Highlights:**

The immune system plays a central role in the pathogenesis of Alzheimer's disease.

The immune system exerts numerous effects throughout the brain on amyloid‐beta, tau, and other pathways.

The 2023 AAIC, Advancements: Immunity, encouraged discussions and collaborations on understanding the role of the immune system.

## 1 INTRODUCTION

Innate and adaptive immunity have emerged as key players in the onset and progression of Alzheimer's disease (AD) and related dementias (ADRD).^1^ Beyond the well‐established pathological hallmarks of AD—extracellular amyloid‐β (Aβ) plaques, and intracellular neurofibrillary tangles (NFTs) composed of hyperphosphorylated tau—researchers have increasingly appreciated the contribution of early neuroinflammation, mediated by microglia and astrocytes, in AD pathology.^2^ Mounting evidence suggests that dysregulation of microglia, the tissue‐resident macrophages of the brain parenchyma and the primary innate immune cells of the central nervous system (CNS), significantly impacts AD pathogenesis. This impact occurs through involvement in critical processes such as neuronal homeostasis, myelin turnover, clearance of extracellular aggregates, synaptic plasticity, synaptic pruning, cellular sensing, T‐cell antigen presentation, and blood‐brain barrier (BBB) function.^3^, ^4^ Reactive astrocytes trigger and interact with microglia and release neurotoxic signals, such as inflammatory cytokines and saturated lipids^5^, ^6^ that accelerate degeneration and tau phosphorylation in neurons.^7^, ^8^ Moreover, adaptive immunity has also been shown to be involved in ADRD pathology through lymphocyte infiltration into the CNS and cross‐talk with CNS innate immune cells.^1^

Though the CNS was historically considered immune‐privileged and separated from the peripheral immune system, it has also become increasingly evident that changes in the peripheral immune system can impact the development of ADRD.^9^ A range of factors that primarily engage peripheral immune responses and promote systemic inflammation, including exposure to pathogens and toxins, gut microbiome dysbiosis, and defects or a loss of immunological tolerance (ie, autoimmunity), may promote or exacerbate various neurodegenerative processes.^9^ Furthermore, genetic mutations or changes in metabolic pathways that drive neurodegeneration can affect both the CNS and peripheral immune responses,^10^ highlighting the importance of studying the interplay between peripheral and central immune systems in the context of ADRD. A deeper understanding of the role of central and peripheral immunity in driving ADRD pathology may help elucidate immune system‐targeted therapeutic strategies to alleviate neurodegeneration.

To review research advancements on the role of innate and adaptive immunity in ADRD, and discuss advances in modeling and therapeutically targeting the immune system in neurodegenerative diseases, the Alzheimer's Association convened a multidisciplinary group of researchers at the Alzheimer's Association International Conference (AAIC), Advancements: Immunity, on March 23–24, 2023. This manuscript provides an overview of the discussions from this conference while highlighting gaps in the field that need to be addressed in future research.

## 2 INNATE IMMUNITY IN ADRD

The innate immune system and neuroinflammation play a key role in AD pathogenesis. In particular, an increased understanding of the central contributions of microglia and astrocytes to this process is crucial and has been driven by the application of genetic, functional genomic, transcriptomic, proteomics, and other research tools.^11^ For example, research has shown that microglia express a unique repertoire of genes not expressed by other brain cells, some of which are also expressed by peripheral macrophages.^12^ Furthermore, many late‐onset AD (LOAD)‐associated genes are expressed highly or exclusively by microglia^13^ and/or astrocytes,^14^ highlighting the importance of this glia in AD pathology. These findings also suggest that identifying causes of microglial and astrocyte dysfunction, genetic predisposition, or earlier life insults such as trauma, infection, or normal aging, could help yield new approaches to prevent and treat AD.

### 2.1 Genetic contributions to innate immune dysfunction in ADRD

Researchers have compared brain tissue from individuals with sporadic and atypical dementia to catalog differences and similarities across disorders, including the changes in immune cells. This approach integrates cell‐specific molecular profiling of human disease tissue, genomics, and bioinformatics to identify various cell states and genetic differences that contribute to disease. The data generated can be used to leverage mouse models and systematically model gene network drivers seen in the disease.^15^ Research suggests that scientists can recapitulate gene expression changes reported in human microglia transitions from early stages of tau pathology to later stages of tau‐driven neurodegeneration. This modeling has allowed the identification of multiple transcriptomically‐defined and distinct types of microglia transition states that shift from an early innate immune phase before neurodegeneration to a delayed immune phase after the onset of neurodegeneration. Partitioning of these microglial transitions to the genetic heritability of three tauopathies—AD, progressive supranuclear palsy (PSP), and frontotemporal dementia (FTD)—revealed shared and distinct disease states of varied cell types (ie, microglia, other glia, neurons, and lymphocytes) and brain regions, with distinct gene regulatory networks defining disorder‐specific states. Moreover, the microglia‐associated neuroimmune modules converge on viral response as a common causal factor. These findings from a weighted gene co‐expression analysis (WGCNA) show that disease‐specific cell states and gene‐regulatory networks are uniquely enriched for microglial‐immune signaling markers and genes, implicating them in causal disease mechanisms of sporadic dementia.^16^

The apolipoprotein E (*APOE*) genotype is the most impactful genetic risk factor for sporadic LOAD, with the *APOE ε*4 variant significantly increasing AD risk compared to the *APOE ε*3 variant in White Western European populations. Recent studies have focused on the role of *APOE* genotype on brain microglial and immune functions.^17^ The transition of microglia from the homeostatic phenotype to disease‐associated microglia (DAM) phenotypes includes a multi‐step process. From a homeostatic phenotype to DAM1 (triggering receptor expressed on myeloid cells 2 [Trem2] independent), there is a signal such as AD pathology, aging, or another trigger to become activated. From DAM1 to DAM2 (Trem2 dependent), Trem2 is required for the final transition. In aging and neurodegenerative diseases, this has been shown to be modulated by differential expression of multiple genes, including upregulation of *APOE* in DAM1 microglia.^18^ The isoform‐dependence of *APOE* genotypes on immunomodulation was investigated by comparing brain tissues from *APOE ε*3 and *APOE ε*4 individuals with AD pathology or normal aging pathology.^19^ Future mechanistic studies will further investigate differences between *APOE ε*3 and *APOE ε*4 microglial neuroinflammatory responses. Important also is an understanding of *APOE* isotype functional changes in astrocytes, which express considerably more *APOE* than other CNS cells. As astrocyte‐derived *APOE*/J lipoparticles are an integral trafficking route for neurotoxic long‐chain free fatty acids,^6^ understanding how *APOE* genetics may also impact astrocyte responses to AD pathology and genetics remains integral. In mice, humanized APOE2/3/4 lines crossed with the P301S tau model previously reported increased astrocyte “reactivity” coincident with increased neurodegeneration,^20^ highlighting the intricate communication between astrocytes, microglia, and neuronal health.

**RESEARCH IN CONTEXT**

1. **Systematic review**: The role of immunity in neurodegenerative diseases, including Alzheimer's and other dementias, is an active and growing area of research. The authors of this manuscript report updates and advances in research presented at the 2023 AAIC, Advancements: Immunity, held in March 2023.
2. **Interpretation**: There have been strides in research identifying the role of immunity in dementia research. This manuscript highlights the research presented at the 2023 AAIC, Advancements: Immunity, including the role of innate and adaptive immunity, central and peripheral immune contributions, therapeutic advances in immunity, lessons from other fields, and more.
3. **Future directions**: Understanding the multifaceted roles of immunity in AD pathogenesis will help develop targeted interventions for AD and advance the field of AD precision medicine.

### 2.2 The role of aging in innate immune dysfunction in ADRD

Various non‐genetic factors can play a role in microglia dysfunction. For instance, aging and neurodegenerative diseases share many hallmarks, including cellular senescence.

Accumulation of cellular senescence has been identified in aging. In the aged brain, senescent cells are heterogeneous and sparsely localized, representing only up to 2% of cells.^21^ Senescent glial cells, including microglia, are present in the brains of AD patients and AD‐relevant animal models of neurodegeneration.^22^ To investigate where and when senescent cells appear during brain aging and neurodegeneration, spatial transcriptomics has been used to map senescent cell types using a library that includes 400 senescence, inflammation, and cell marker genes based on previously published senescence signatures.^23^ This analysis showed that senescent microglia were identified sparsely in the aging animal brains and numerously in the tau MAPT P301S PS19 transgenic mouse line. Some microglia exclusively expressed the DAM signature while others co‐expressed both DAM and senescent signatures, suggesting that there are different triggers of DAM and senescent signatures. Ongoing studies are investigating senescence in other CNS cell types trying to understand how these may contribute to AD and other neurodegenerative diseases. Important to these efforts are comparisons between mechanistic rodent studies and correlations possible using human postmortem brain samples. An important question to unravel is what the function of senescent cells is, and what influence they may have on their surroundings.

Elements within the cell, such as lysosomes, can also contribute to microglia dysfunction. Lysosomes play essential roles in cellular metabolism and clearance through coordinated lysosome‐to‐nucleus signaling. The transcription factor EB (TFEB)—a master regulator of genes involved in lysosomal biogenesis and function as well as a broad range of other targets—mediates the degradation of cellular organelles and long‐lived proteins such as tau and NFTs.^24^, ^25^ Investigating the specific role of lysosomal TFEB in AD pathogenesis by manipulating the vacuolar ATPase (v‐ATPase) transcriptional program has further demonstrated that the lysosomal TFEB pathway is essential for maintaining lysosomal pH and homeostasis under physiological conditions and the induction of microglia activation in response to tau pathology.^26^ In addition, single‐cell pathway analysis identified metabolic signatures that suggest impaired metabolic responses of lysosomes in microglia to pathological conditions.^26^

## 3 ADAPTIVE IMMUNITY IN ADRD

In addition to the role of innate immunity, recent studies have implicated a role for the adaptive immune response in ADRD. The role of adaptive immunity in AD pathology has been suggested by animal studies involving the depletion of T cells, B cells, and NK cells.^1^ Furthermore, accumulating evidence suggests T‐cell infiltration in the CNS promotes neurodegeneration and functional decline in AD, but what causes this infiltration is not well understood. Several studies have sought to characterize the molecular mechanisms involved in T‐cell infiltration, link T cells to distinct hallmark pathological features of AD, and identify T‐cell populations in the presence of various antigens in the context of AD.^27^

### 3.1 Role of T cells in AD

T cells have been implicated in both the pathogenesis and prevention of AD pathologies in preclinical animal models, but the T cell subtypes and cytokines involved in either pathway remain unclear. Research conducted by various groups focusing on specific subtypes of CD4+ T cells in AD mice has led to divergent findings. For instance, a study depleting regulatory CD4+ T cells (Tregs) in the amyloid 5xFAD mouse model demonstrated a reduction in pathology,^28^ whereas another study involving Treg depletion in the 3xTg‐AD mouse model reported increased Aβ plaques in the hippocampus associated with a marked aggravation of the spatial learning deficits of the treated mice.^29^ These conflicting results may arise from different AD models and artifacts related to transgene expression,^30^, ^31^ disease progression stages, criteria for pathological assessment, or techniques employed for inducing or depleting Tregs. However, they may also indicate a nuanced interplay between AD pathology and immune tolerance. Recently, in a tauopathy mouse model, infiltrating T cells promoted microglia‐mediated cell death in the CNS, and depletion of these T cells alleviated this cell loss.^32^ However, enhancing the response of Tregs in the brain through checkpoint blockade also reduced microglial reactivity and neurodegeneration,^32^ highlighting the disparate roles different T‐cell subtypes can play in AD pathology.

Several factors, such as aging, genetics, cellular senescence, and pathogen exposure, that influence AD progression and onset may also directly impact the T‐cell repertoire.^33^ Cellular senescence is associated with telomere shortening, and a common feature is senescent‐associated B‐galactosidase (SA‐β‐gal) activity.^34^ Senescent CD8+ T cells express higher SA‐β‐gal activity in older individuals compared to younger individuals, and this was observed in African Americans and non‐African Americans.^35^ Interestingly, high levels of senescent CD8+ T cells correlate with decreased performance in the acquired equivalence task, decreased physical activity, and poorer VO_2_ max scores as described at the conference,^36^ thus suggesting a relationship between immune senescence and physical activity and subsequently between immune senescence and AD risk factors. Future studies need to evaluate the relationship between other AD‐associated markers with immune cell senescence profiling, with particular emphasis on mitochondrial dysregulation and CD8+ T‐cell subsets in aging as well as the function of senescent cells.

Another example is during aging, senescent and exhausted T cells exhibit loss of CD27 and CD28 costimulatory molecules, decreased telomerase activity, reduced immune responsiveness, increased susceptibility to infections, and decreased T‐cell receptor repertoire.^37^ In addition, exposure to antigens from viruses or bacteria may lead to T‐cell exhaustion and secretion of cytotoxic cytokines, which can be particularly detrimental in AD brains.^33^ Polymorphisms in the human leukocyte antigen (*HLA*) gene can lead to differential processing and presentation of antigens to T cells, which could confer differential immune responses in the context of AD. Furthermore, T‐cell responses to self‐antigens—including Aβ and tau—and microbial non‐self‐antigens may exacerbate T‐cell responses in AD.^38^

### 3.2 Peripheral T‐cell alterations and preclinical AD

To investigate whether peripheral adaptive immune cell alterations reflect early changes in AD biomarkers, peripheral blood mononuclear cells of 251 participants (cognitively healthy, or those with mild cognitive impairment or probable AD) were immunophenotyped in cross‐sectional and longitudinal studies. Using multidimensional mass‐cytometry combined with unbiased machine‐learning techniques, researchers have recently shown that increased levels of Aβ in the brain and changes in plasma AD biomarkers were associated with an increase in antigen‐experienced adaptive immune cells in the blood, particularly CD45RA‐reactivated T‐cell effector memory (TEMRA) cells, even in cognitively healthy subjects. These data suggest that peripheral changes in adaptive immune cells may be used as a proxy for CNS biomarker changes.^39^

### 3.3 Microglia‐mediated T‐cell infiltration and reactivity

Microglia may drive the link between adaptive immunity in AD pathology by mediating T‐cell infiltration in the CNS, which is further exacerbated by tau‐mediated neurodegeneration.^1^, ^32^ Immune single‐cell RNA profiling showed that a T‐cell population is strongly increased in the brains of mice with tau pathology but not in those with Aβ deposition. Infiltrated T‐cell numbers as well as tau pathology were reduced on microglia depletion. This suggests a pivotal role for microglia, in the setting of a tauopathy‐specific immune environment by recruiting T cells into the brain parenchyma and a detrimental role. It was further shown that microglia are required for T‐cell infiltration in the brain by regulating the interferon response and antigen‐presenting features. T cells correlated with the extent of neuronal loss and dynamically transformed their cellular characteristics from activated to exhausted states along with unique T‐cell receptor clonal expansion.^1^, ^32^, ^40^ Together, these data suggest that neuronal loss may be due in part to T‐cell infiltration mediated by microglia in the presence of tau.

Microglia are antigen‐presenting cells in the CNS and express human leukocyte antigen–DR isotype (HLA‐DR),^41^ which may be important for driving neuroinflammation. Among many genetic variations, a single nucleotide polymorphism (SNP) in the non‐coding region in HLA‐DR is associated with late‐onset sporadic Parkinson's disease (PD), and expression is elevated in sporadic and familial PD patients.^42^ HLA‐DR (or MHCII in mice) expression is also correlated with brain CD4+ and CD8+ T‐cell infiltration in human postmortem tissue and a mouse model of PD.^43^ T cells recognize and respond to α‐synuclein peptides which have a high affinity for binding to two HLA alleles.^44^ Alpha‐synuclein is implicated in PD risk and researchers have demonstrated that α‐synuclein promotes microglia antigen processing and presentation, CD4 T‐cell activation, and proliferation in vitro and in vivo.^45^ Overexpression of human α‐synuclein in neurons of a PD murine model increased MHCII expression and T‐cell (CD4+) infiltration.^46^, ^47^ Moreover, MHCII expression on CNS‐resident macrophages drives CD4+ T‐cell infiltration and neurodegeneration. Blocking MHCII expression or CD4 T cells attenuates α‐synuclein‐mediated inflammation and neurodegeneration, and thus demonstrates that the interaction between antigen‐presenting cells and CD4+ T cells is required for dysregulated immunity that occurs during neurodegeneration.

Pathogen infection of microglia activates many immune signaling pathways which may be involved in recruiting T cells to the CNS. Herpes simplex virus type 1 (HSV‐1) is of particular interest, as it has been implicated in AD development and is a risk factor in individuals who are *APOE*4 carriers. LOAD genetic variation may modulate the microglia response to HSV‐1, but studies of this require a model for HSV‐1 microglia infection. To address this, researchers generated human microglia‐like cells (MDMi) and infected them with HSV‐1. HSV‐1‐infected MDMi cells had higher expression of interleukin (IL)‐1β, tumor necrosis factor (TNF)‐α, and interferon‐stimulated gene 15 (ISG15) that was time‐ and dose‐dependent.^48^ Furthermore, HSV‐1 infection of MDMi cells induced CXCR3+ CD8+ effector T cells but led to a decrease of cytotoxic CD8+ T cells expressing granzyme B.^49^ Future studies are focused on understanding the role of microglial genetics on MDMi response to HSV‐1 infection and the T‐cell response.

### 3.4 *APOE* and T‐cell regulation

*APOE* genotypes lead to differentially expressed (DE) genes that may lead to the development of AD. Furthermore, these *APOE* genotypes regulate epigenetic changes, specifically differential accessibility (DA) in the chromatin. Monocytes, B cells, and CD8+ T cells in the periphery of AD patients have more accessible chromatin compared to healthy controls. *APOE ε*4/*ε*4 monocytes in AD patients have the most DE genes compared to healthy controls. The overlap between DA associated with a DE gene is of particular interest because chromatin accessibility is a key factor influencing gene expression. Monocytes have the most overlapping genes followed by T cells and NK cells. Cytokine genes found in the overlap region of AD monocytes are IL‐β, CCL4L2, and CCL3. Notably, the transcription factor binding sites in *APOE ε*4/*ε*4 carriers are enriched but not in *APOE ε*3/*ε*3 carriers. Thus, *APOE ε*4/*ε*4 CD8+ T cells have increased accessibility and DE genes in AD compared to healthy controls. Importantly, AD risk genes from genome‐wide association studies (GWAS) are also expressed by peripheral immune cells and have differentially accessible chromatin regions in AD.^50^

## 4 CENTRAL AND PERIPHERAL IMMUNE CONTRIBUTIONS TO NEURODEGENERATION

Peripheral immune cells communicate and modulate brain function during homeostasis and AD progression. Furthermore, peripheral immune insults or dysregulation may contribute to cognitive and functional decline in AD.^51^ However, there are no consistent data suggesting that peripheral immune mechanisms directly drive AD pathogenesis. A multi‐platform proteomic analysis of AD cerebrospinal fluid (CSF) and plasma that are associated with proteostasis and the matrisome revealed CNS pathways and lack a robust peripheral immune signature.^52^ Therefore, the absence of peripheral biomarkers could be an indicator that the peripheral immune system is not directly involved in driving AD pathogenesis. Furthermore, most immune markers in the CSF reflect changes in the CNS but not peripheral changes. Genes or candidate genes for AD risk such as *TREM2*, *SPP1*, *APOE*, *INPP5D*, and *PLCG2* that affect immune function are expressed by immune cells in the brain.^53^, ^54^, ^55^, ^56^, ^57^ Finally, research shows that peripheral immune cells do not directly affect neurons and synapses but rather interact with brain‐resident immune cells such as microglia, border‐associated macrophages (BAMs), and glia (astrocytes/oligodendrocytes).^3^, ^32^, ^58^, ^59^, ^60^ Consistent, reproducible data are needed to fully implicate the peripheral immune system as a direct driver of AD pathology. However, crosstalk between the peripheral immune system and the CNS may contribute to cognitive decline in AD.

### 4.1 Crosstalk between the peripheral and CNS immune systems

Research shows substantial crosstalk between the peripheral immune system and the CNS during homeostasis and disease. This crosstalk includes indirect communication mediated through cytokines as well as physical infiltration of peripheral immune cells into the CNS.^51^

#### 4.1.1 Cytokines

Numerous peripheral cytokines (eg, IL‐4 and IL‐17), many of which are produced by meningeal T cells, can communicate with the CNS and contribute to cognitive function. For example, IL‐4‐producing T cells accumulated in the meninges of mice performing cognitive tasks, and loss of IL‐4 resulted in cognitive defects.^61^ In addition, IL‐17 derived from meningeal γδ T cells has been shown to control synaptic plasticity and short‐term memory.^62^ Depletion of IL‐17 rescued cognitive impairment and synaptic dysfunction in an AD mouse model.^63^

IFN‐γ expression leads to age‐progressive midbrain pathologies in mice.^64^ IFN‐γ stimulation of monocytes and T cells results in the upregulation of numerous interferon‐stimulated genes, including leucine‐rich repeat kinase 2 (LRRK2).^65^ Genetic mutations in LRRK2, including the G2019S mutation, cause autosomal dominant PD,^66^ but whether LRRK2 G2019S and IFN‐γ synergize to accelerate the development of a PD phenotype remains unclear. Expression of IFN‐γ in neonatal LRRK2 G2019S knock‐in mice^67^ led to increased tau phosphorylation on specific epitopes in the cortex and midbrain, potentially explaining why some LRRK2 G2019S PD patients present with tau pathology.

#### 4.1.2 T cells

T cells themselves also mediate physical communication between the periphery and the CNS. In mice, CD4+ T cells infiltrating the CNS are required to drive the maturation of microglia.^68^ Additionally, infiltrating peripheral T cells contribute to the maintenance of neurogenesis and spatial learning abilities in adult mice.^69^ Throughout aging, T‐cell infiltration into different areas of the mouse brain increases. These T‐cell infiltrates modify the inflammatory profiles of microglia and oligodendrocytes^70^ and decrease neural stem cell proliferation.^71^

#### 4.1.3 Macrophages

Macrophages and monocyte‐derived cells can also infiltrate the CNS during certain disease states. Recently, peripheral disease inflammatory macrophages (DIMs) that are distinct from CNS‐resident DAMs have been identified in aged brains with Aβ plaques.^72^ Depletion of peripheral monocyte‐derived cells via splenectomy leads to an increase in Aβ plaques,^73^ and C‐C chemokine receptor type 2 (CCR2)‐expressing perivascular macrophages have been shown to clear Aβ plaques.^74^

#### 4.1.4 Sepsis mouse models

Sepsis is characterized by multi‐organ dysfunction following unresolved infections leading to a dysregulated immune response.^75^ Elderly sepsis survivors are over three times more likely to develop severe cognitive impairment compared to elderly, non‐septic individuals, suggesting that systemic inflammation associated with sepsis may modify AD pathogenesis.^76^ Modeling of sepsis in rodents has been complicated by the lack of standardization across protocols for inducing sepsis as well as the environment and age of the animals used.^75^, ^77^ Most sepsis models in mice use lipopolysaccharide‐induced endotoxicosis, thereby not capturing the dual inflammatory and immunosuppressive phenotypes observed in sepsis patients.^78^ As a result, studies of AD and sepsis in animal models have produced conflicting results. Despite this, some studies have shown that sepsis can trigger synapse loss, and spatial memory defects, as well as influence Aβ deposition, although the degree and severity depend on the model.^75^, ^79^ However, how sepsis‐induced peripheral immune system changes impact the CNS in these models remains unclear.

To directly examine how changes in peripheral immunity influenced the CNS, a recent study induced sepsis in an early‐onset AD mouse model using cecal ligation and puncture combined with daily chronic stress for 7 days.^80^ A sex‐dimorphic and transgene‐specific alteration was observed in splenic hematodysplasia. Further, mice that were aged following recovery from sepsis accumulated a higher Aβ burden. Sepsis induced a sex‐dependent increase in astrocyte proliferation in all animals, but brain‐resident microglia in AD mice failed to proliferate. These CNS changes resulting from sepsis suggest that infection in the periphery may play a role in AD pathogenesis.^81^

## 5 IMPROVED MODELING OF IMMUNE RESPONSES IN PRECLINICAL MODELS

Characterizing immune system contributions to AD requires the continued development of appropriate cellular and animal models. In particular, model systems for studying the effect of the peripheral immune response on the CNS should mirror human disease courses and be standardized across the field. In addition, genetic factors relevant to these immune system contributions—and that reflect the broader complexity of AD—must be reflected in animal models.^82^ Various heritable risk alleles in the human genome are associated with AD development, and frequently, multiple genes contribute to the disease. Several mouse models that have made progress in achieving these goals were presented at the conference and are highlighted below.

### 5.1 Mouse models of Alzheimer's disease risk alleles

Mouse models that express human AD genetic risk alleles are important tools for studying the pathogenesis of AD.^83^, ^84^ The Model Organism Development & Evaluation for Late‐Onset Alzheimer's Disease (MODEL‐AD) consortium has developed numerous mouse models based on risk alleles for LOAD. Mice are genetically modified based on the risk allele of interest, then aged and conditioned with the appropriate environmental stimuli such as a high‐fat diet to accelerate induced neurodegeneration.^85^ These models are comprehensively characterized with all the models and data available without licensing restrictions, and data can be used for the evaluation of potential therapeutics.^86^, ^87^ One example presented at the conference is a mouse model of the *Inpp5d*, which encodes inositol polyphosphate‐5‐phosphatase D/SHIP1.^88^ Specifically, many DE genes associated with neurodegeneration were identified in the microglia of *Inpp5d*‐deficient mice, and this differential gene expression was more pronounced in female mice.^89^ In particular, previous use of spatial transcriptomics to investigate *Cx3cr1‐dependent* deletion of *Inpp5d*, when crossed with APP/PS1 amyloidosis mice, highlighted an extended gene expression signature associated with plaques and identified CST7 (cystatin F) as a novel marker of plaques.^90^

### 5.2 Humanized mouse models

While rodent models are important tools for studying AD, rodents do not recapitulate all human AD pathologies due to numerous genetic and cellular differences. These differences, as well as lifespan and environmental differences, contribute to the discrepancy between rodent and human immune responses.^91^ Mouse models that incorporate human cells allow for the study of human cells and genetics in an experimentally manipulatable system. For example, the microglia in vitro generation refined for advanced transplantation experiments (MIGRATE) protocol is used to transplant human microglia into mice.^92^ Human induced pluripotent stem cells (iPSCs) are cultured, differentiated into microglia, and then transplanted into the brains of immunodeficient mice. In addition, a human *CSF1R* variant has been engineered to provide a nontoxic microglial replacement, suggesting the potential therapies involving the delivery of tissue‐resident macrophages as living therapies to modulate microglial function and genetics in the diverse neuropathologies.^93^, ^94^

Despite the advantages of mouse models for studying immunity and AD, many orthologues of human and mouse AD risk genes share limited homology that limits opportunities to explore the therapeutic potential in the models. To address these interspecies differences, studies have investigated the extent to which mimicking the developmental ontology of microglia in vitro can help differentiate human iPSCs into microglia.^95^, ^96^, ^97^, ^98^, ^99^, ^100^, ^101^, ^102^, ^103^ However, because microglia cultured in vitro containing serum exhibit rapid changes in RNA expression^104^ (though it should be noted this can be minimized with omission of serum and use of defined trophic culture media^105^) researchers investigated whether in vivo transplantation of human iPSCs into mouse brain might induce a more human‐like microglial phenotype. Notably, this approach requires immune‐deficient mice, thus creating a microglial model independent of T‐cell interactions. Validation studies show that, indeed, transplanting human microglia progenitors into neonatal mouse forebrain promotes the adoption of an in vivo‐like microglial transcriptome and microglial functions. In addition, transplantation in the 5XFAD AD model revealed characteristic microglial migration toward Aβ plaques and broad changes in gene expression toward a DAM‐like phenotype. Notably, however, expressed human and mouse DAM genes overlapped by only 10%, providing clear evidence for species differences in microglial responses. Chimeric models can also be combined with CRISPR editing to examine the impact of other AD risk genes (eg, *TREM2*).^106^

Transplantation of human iPSC‐microglial progenitors is also being developed for therapeutic applications. As proof‐of‐principle, using CRISPR engineering for regulated delivery of neprilysin, an Aβ‐degrading enzyme, in an AD mouse model demonstrated reduced Aβ pathology and astrogliosis and protection from the loss of synaptic proteins. Ongoing studies are focused on scaling up this approach to improve microglial‐payload delivery in the adult brain.^94^

### 5.3 Marmoset models of Alzheimer's disease

Non‐human primates, like marmosets, undergo age‐dependent changes in motor and cognitive function, spontaneously present Aβ plaques and neurofibrillary tangles, have high genetic homology with humans, and can be genetically manipulated.^107^, ^108^ Additionally, there is significant homology between marmoset and human immune responses.^109^ As proof of concept, *PSEN1* mutations identified in human AD (C410Y and A426P) were introduced into marmosets.^108^, ^110^ Cognitive and behavioral assessments coupled with measures of fluid biomarkers as well as multi‐omic analyses will be used to track AD pathogenesis in marmosets.^108^, ^111^ Notably, marmoset models could be used to identify early molecular determinants of AD pathogenesis prior to symptom onset and frank neuropathology.

## 6 BIOMARKERS FOR IMMUNE CELL CHANGES IN THE BRAIN

AD therapeutics development should include interventions that consider and potentially harness the peripheral immune system. Designing appropriate therapeutics requires the identification and longitudinal tracking of biomarkers in the periphery and CNS to identify potential drug targets and assess therapeutic efficacy, as well as an understanding of the role of these biomarkers in diverse populations. Progress on both goals has pointed the way toward promising therapeutics targeting the immune system.^112^

### 6.1 Free water in the brain as a biomarker to assess inflammation

The use of immune modulators to treat AD requires assessment of changes in inflammation‐associated biomarkers such as free water in the brain.^113^ Healthy, myelinated axons are surrounded by free water, and in AD patients, there is an increase in free water levels.^113^ Research presented at the conference indicated that free water levels as measured by white matter neuroimaging correlated with a variety of AD biomarkers, CSF inflammatory proteins, and worsening cognition. Free water measurements, coupled with measurements of inflammation in the CSF and white matter microstructural changes, indicated that xPro1595, a next‐generation selective inhibitor of soluble TNF, decreased neuroinflammation while improving white matter measures of apparent fiber density and radial diffusivity in a Phase 1b trial of AD patients (NCT05522387).

### 6.2 Immune‐related plasma proteins and microbiome biomarkers of AD pathology

Systemic health—particularly immunologically relevant conditions, such as autoimmune or inflammatory disease—are known to influence the risk of AD and other dementias.^114^ Researchers suspect that proteins circulating in the blood likely mediate this link between systemic health and dementia risk.^115^, ^116^ To ascertain which blood proteins may increase AD risk, researchers can now make use of protein quantitative trait loci (pQTLs) recently made available as a result of the broad implementation of high‐throughput proteomic platforms and genetic analysis on a population level.^117^ Using identified genetic loci that code for blood protein abundance, researchers can now use observational data in aging cohorts to predict which plasma proteins likely have a mechanistic relationship with AD and related phenotypes. Studies using this approach to identify putative causal proteins in blood have consistently implicated immune‐related proteins, including SERPINA3 and SVEP1 as peripheral drivers of AD risk.^116^, ^117^, ^118^

Another important component of systemic health is the microbiome, and recently the microbiota‐gut‐brain axis has gained attention in Alzheimer's research. Studies suggest that changes in gut microbiota are linked to AD progression, with various proposed mechanisms.^119^ Fecal transplant from healthy to AD mice has been shown to reduce Aβ plaques in the brain,^120^ and this clearance has been associated with lower levels of *Bacteroides fragilis*.^121^ To determine how *B. fragilis* affects the immune‐mediated clearance of Aβ plaques, *B. fragilis was* administered to AD‐predisposed mice. *B. fragilis* exposure resulted in an increase in Aβ plaques in the brain of AD mice as well as reduced uptake of Aβ peptides by microglia.^122^

### 6.3 Mouse models of protein biomarkers

Classic AD biomarkers include Aβ plaques and tau tangles, but other proteins have also been established as potential biomarkers, including TAR DNA‐binding protein 43 (TDP‐43) and chitinase‐3‐like protein 1 (CHI3L1).^123^ Despite their status as biomarkers, their contribution to disease pathology remains unclear. Manipulating TDP‐43 and CHI3L1 expression in mouse models can help elucidate their roles in disease and reveal potential therapeutic opportunities.

TDP‐43 is a ubiquitously expressed nucleic acid‐binding protein localized predominantly in cell nuclei under normal physiological conditions.^124^, ^125^, ^126^, ^127^ However, studies have identified extranuclear TDP‐43 in neurons and glial cells, including oligodendrocytes and astrocytes, in various neurological disorders, including AD.^128^, ^129^, ^130^, ^131^, ^132^, ^133^ For example, recent work suggests that TDP‐43 can be mislocalized in hippocampal astrocytes in AD and FTD. In mice, TDP‐43 mislocalization in hippocampal astrocytes resulted in increased neuroimmune signaling and interferon‐related gene expression, including leading to increased levels of astrocytic C‐X‐C motif chemokine ligands 9 and 10 (CXCL9 and CXCL10) as well as their receptor, CXCR3, in excitatory presynaptic terminals. Increased CXCR3 signaling altered excitatory transmission that contributed to memory deficits.^134^ Additionally, astrocyte‐specific TDP‐43 mislocalization led to increased expression of antiviral factors, including cytosolic double‐stranded RNA (dsRNA) sensors, and cell‐autonomous susceptibility to viral pathogens, including HSV‐1.^134^ These findings suggest a model in which astrocytic TDP‐43 dysregulation contributes to pathogenesis in AD and FTD through alterations in neuroimmune signaling and viral susceptibility, potentially due to aberrant host RNA processing.

CHI3L1 functions in the periphery as a signaling mediator for a range of immune responses.^135^ In humans, CHI3L1 is predominantly expressed in astrocytes.^136^ In mice, Chi3l1 is equally expressed by both astrocytes and oligodendrocytes and global overexpression of Chi3l1 drives subsequent increased chemoattractant receptor homologous molecule expressed on T helper type 2 cells (CRTH2) signaling in neuronal stem cells (NSCs) causing decreased NSC proliferation.^137^ Reduced NSC proliferation can lead to impaired adult hippocampus neurogenesis and decreased cognitive performance. Because depletion of CHI3L1 can rescue this phenotype, CHI3L1 may be a promising therapeutic target for AD.^137^

## 7 CLINICAL TRIALS AND EMERGING PERIPHERAL THERAPEUTICS IN DEMENTIA

Potential AD therapeutics that leverage the peripheral immune system include preexisting drugs such as antivirals, immunization targeting Aβ, and novel methods to induce differentiation of Aβ‐targeting immune cells.

### 7.1 Diversity and inclusivity in clinical trials

Traditionally, AD clinical trials and biomarker identifications have been conducted in non‐Hispanic Whites. However, a meta‐analysis demonstrated that the AD rate for Black/African American adults was 64% higher than for Whites.^138^ Despite this, Black/African Americans are underrepresented in clinical trials for AD, and current data lack information on AD biomarkers in Black/African Americans. To address this gap, researchers enrolled healthy middle‐aged non‐Hispanic Whites and Black/African Americans at risk for AD in a longitudinal study (ASCEND Study) to investigate the interplay between AD biomarkers, brain and systemic inflammation, and AD development. Early analysis indicates that Black/African Americans have lower levels of tau but higher pro‐inflammatory markers in the blood compared to non‐Hispanic Whites, indicating biomarkers differ between populations.^139^

### 7.2 Antiviral treatment

HSV‐1 is detected in the brain of AD patients at higher rates than in cognitively normal patients and is associated with worse cognition.^140^ In mice, HSV‐1 infection induces Aβ and tau formation; administering antiviral drugs can protect against these effects.^141^ In a pilot trial of 33 patients, valacyclovir 3 gm daily was well‐tolerated and showed measurable changes in CSF levels of inflammatory markers.^142^ Two ongoing controlled clinical trials aim to assess the effects of valacyclovir versus placebo in HSV seropositive patients with mild to moderate AD (VALAD) or mild cognitive impairment (VALMCI).^143^ Patients will be evaluated for changes in cognition, function, and biomarkers such as Aβ and tau via positron emission tomography (PET) imaging.

### 7.3 Aβ immunization

Clinical trials targeting Aβ have been under investigation for over two decades,^144^ highlighting their potential as a promising therapeutic approach in AD. Initial evidence of their efficacy emerged from postmortem analyses of the AN1792 active Aβ immunotherapy trial. These analyses demonstrated Aβ clearance in a subset of patients, irrespective of dementia progression.^145^, ^146^, ^147^, ^148^, ^149^ This finding prompted a series of critical questions regarding the cellular mechanisms responsible for Aβ removal and the precise role of Aβ in the progression of dementia.^146^

### 7.4 Cell transplantation

T cells targeting AD‐associated proteins like Aβ precursor protein (APP) could help restrict AD pathology. However, during development in the thymus, T cells that are specific to APP expressed on thymic epithelial cells (TECs) may be pruned to eliminate self‐reactive T cells. Additionally, aged AD mice have reduced T cell generation because of enhanced loss of TECs compared to non‐AD aged mice. To combat this, researchers supplemented these mice with APP‐expressing or APP‐depleted TECs. Transplantation of APP‐competent or APP‐depleted TECs into mice attenuated AD pathology, but APP‐depleted TECs exhibited greater effectiveness, leading to an increase in Aβ‐specific T cells. Transplantation of APP‐depleted human TECs is a potential therapy for AD patients.^150^

## 8 CROSS‐DISEASE INSIGHTS FOR AD

Dysregulated cellular interactions between immune and non‐immune cells in the CNS and between the CNS and the peripheral immune system may contribute to AD pathology. Thus, targeting proteins that regulate these cellular networks and generally treating diseases that trigger dysregulated immune interactions may be viable therapeutic strategies. However, therapeutic development first requires further elucidation of both the protein and cellular networks and diseases involved. Lessons from other fields offer insights into potential directions for future research.

### 8.1 Cellular communication in the CNS

Cell‐cell interactions control CNS physiology and pathology, and further development of unbiased methods is needed to holistically study the complexity of cell‐cell communication. Recent studies have developed two methods for studying cell interactions in the CNS—rabies barcoding in droplets followed by sequencing (RABID‐seq)^151^ and stimulation, perturbation, and encapsulation of interacting cells followed by sequencing (SPEAC‐seq).^152^

RABID‐seq involves injecting barcoded rabies viruses into the brains of mice and using these barcodes to reconstruct cell interactions with single‐cell RNA sequencing.^151^ This method, used to examine the role of semaphorin 4D (SEMA4D) in an experimental autoimmune encephalomyelitis (EAE) model of multiple sclerosis (MS) has provided crucial insights into the role of inflammation in neurodegeneration which are relevant to AD pathology as well. RABID‐seq analysis indicated that SEMA4D on microglia interacted with receptors on astrocytes, leading to increased inflammation and neurodegeneration. SEMA4D was found to be upregulated in neurons during Huntington's disease (HD) and AD disease progression. SEMA4D normally regulates the actin cytoskeleton and inflammatory transformation through cognate receptors expressed on glial cells, and during neurodegeneration increases astrocyte reactivity and inhibits their normal homeostatic metabolic functions.^153^ These findings led to a trial of an SEMA4D antibody (pepinemab) in HD patient cohorts, which restored deficits in metabolic activity as measured by fluorodeoxyglucose (FDG)‐PET and delayed cognitive decline.^154^ This example illustrates how insights from MS and HD research can inform therapeutic strategies for AD.

Further screening of cell interactors can be done with SPEAC‐seq. In SPEAC‐seq, one cell of interest (eg, microglia) is transduced with a CRISPR/Cas9 library while the target cell (eg, astrocyte) is transduced with a fluorescent reporter that is expressed after activation of a target gene (eg, NF‐κB). The cells are then co‐cultured in droplets followed by quantification of the fluorescent reporter to develop a catalog of genes in the cell of interest that signal to the target cell to induce expression of the target gene. SPEAC‐seq was recently used to determine that astrocyte‐derived IL33 induces amphiregulin (*Areg*) expression in microglia which in turn decrease NF‐kB signaling and minimized astrocyte reactivity in EAE, indicating IL33‐AREG‐NF‐kB signaling controls an astrocyte–microglia regulatory circuit.^152^

### 8.2 Peripheral communication with the CNS

Genetic mutations that drive disease are not restricted to cells in the brain, and systemic changes resulting from these mutations may influence disease pathology. For example, research on heterozygous loss‐of‐function mutations in granulin (GRN) associated with frontotemporal lobar degeneration (FTLD) has shown how peripheral immune responses can affect CNS pathology, suggesting potential therapeutic targets for AD. In the CNS, the depletion of GRN from mice resulted in increased synaptic pruning by microglia.^155^ In aged GRN‐deficient mice, less reactive monocytes infiltrated the brain at higher levels which was coupled with an increase in peripheral immune responses.^156^ Thus, targeting myeloid cell trafficking into the CNS could be a potential therapeutic target to alleviate neurodegeneration.

Inflammatory signaling from both the periphery and the CNS plays a critical role in the progression of neurodegenerative disorders.^157^ The fruit fly, *Drosophila melanogaster*, is a model organism equipped with genetic and physiological tools to study the contributions of peripheral and CNS inflammatory signaling pathways in the context of neurodegenerative diseases. These techniques enable the development of humanized flies to model disorders such as PD and AD, providing a platform to study cellular mechanisms and potential therapeutic targets to mimic PD pathology; human alpha‐synuclein (hSNCA) and mutant hSNCA were expressed in either the brain or the periphery of *Drosophila*. Expression of both wild‐type and mutant hSNCA in the brain or gut resulted in phosphorylation and accumulation of hSNCA in the brain^158^ and led to behavioral deficits that include motor activity deficiencies and sleep disturbances. Gut expression of hSNCA was associated with a significant increase in TNF and Toll receptor signaling proinflammatory markers.^158^ Future studies using this physiological platform will further investigate the cellular mechanisms that lead to neuronal dysfunction and behavioral symptoms resulting from hSNCA expression in peripheral organs or the brain.^158^

### 8.3 CNS metabolism

Quantitative proteomics of brain and CSF samples have indicated a strong relationship between AD pathology and metabolic pathways associated with microglia and astrocyte reactivity.^159^ Many of these pathways are regulated by insulin signaling, suggesting insulin treatment may be a potential AD therapy. For example, the repurposing of insulin enhances immunometabolism in the brain^160^ and counter‐regulates tau pathologies.^161^ In a recent Phase IIB clinical trial, intranasal insulin (INI) delivered to the brain of AD patients^162^ improved AD biomarker profiles and inflammation^163^ while slowing vascular damage indicated by slowed white matter hyperintensity progression.^164^

### 8.4 Autoimmunity

An increased understanding of autoimmune disorders associated with AD may help identify mechanisms of how an overactive immune system influences AD pathogenesis.^165^ Data from electronic medical records (EMRs) was used to determine if autoimmunity increased the odds of developing AD. Diagnosis of an autoimmune disease, especially one related to the endocrine, hematologic, and musculoskeletal systems, increased the odds of an AD diagnosis while decreasing the time to that diagnosis.^166^

Increased prevalence of autoimmunity has also been associated with other neuropathologies, including concurrent amyotrophic lateral sclerosis (ALS) and MS.^167^ All patients with concurrent ALS and MS had a mutation in C9orf72, a protein involved in lysosomal trafficking.^167^ To determine whether C9orf72 contributed to both autoimmunity and neurodegeneration, researchers generated C9orf72‐deficient mice. Some C9orf72‐deficient mice spontaneously developed a fatal autoimmune phenotype.^168^ This phenotype was associated with enhanced type‐I IFN signaling in DCs caused by delayed STING degradation.^169^ Aged C9orf72‐deficient mice exhibited enhanced synapse loss, complement deposition, and memory deficits as well as systemic inflammation.^170^ Paradoxically, C9orf72‐deficient microglia were better at clearing plaques despite promoting increased synaptic damage. This suggests that genetic mutations associated with the development of autoimmune disorders may also play an important role in neuropathologies.

## 9 CONCLUSION

The biomedical research community has made significant progress in understanding the role of immunity in the development of AD and other neurodegenerative diseases. The 2023 AAIC, Advancements: Immunity, helped to facilitate discussions on emerging research in immunity and ADRD and provide a forward perspective on the field. Sessions highlighted the role of the innate and adaptive immune system in neurodegenerative diseases, animal models of immunity in AD that replicate human pathology, immune‐related biomarkers, clinical trials, and lessons from other fields describing the role of the immune system in neurodegeneration. Understanding the role of immunity in AD and other dementias requires further attention to bidirectional communication between the brain and periphery. While human studies have been correlative and have not demonstrated the direct involvement of the peripheral immune responses in AD pathologies, there have been a number of tightly controlled animal studies that show mechanisms and the crosstalk between the periphery and CNS suggesting that various aspects of the peripheral immune system may be potential therapeutic targets for AD.^51^, ^115^

While the 2023 AAIC, Advancements: Immunity, highlighted research on the role of immunity in ADRD, it also demonstrated areas in which further work is needed to clarify how the components of the immune system contribute to either disease progression or protection. Delineating the roles of key CNS immune cells as well as elucidating how peripheral inflammation affects the CNS requires models that reflect human immune system complexity and techniques incorporating lessons from other fields. Understanding how the immune system modulates AD pathogenesis will be essential for developing therapeutics harnessing either the central or peripheral immune response to treat neurodegenerative disease.

## CONFLICT OF INTEREST STATEMENT

C. M. Kloske is an employee of the Alzheimer's Association. S. Mahinrad is an employee of the Alzheimer's Association. C. J. Barnum is an employee of INmune Bio, Inc. A. F. Batista has nothing to disclose. E. M. Bradshaw has nothing to disclose. B. Butts has nothing to disclose. M. C. Carrillo is an employee of the Alzheimer's Association. P. Chakrabarty has nothing to disclose. X. Chen has nothing to disclose. S. Craft has served as a Scientific Advisory Board member for T3D Therapeutics, Inc. and for the Neurodegeneration Consortium. S. Da Mesquita was listed as an inventor in patent applications concerning meningeal lymphatic function in neurological diseases. L. C. Dabin has nothing to disclose. D. Devand has nothing to disclose. V. Duran‐Laforet has nothing to disclose. W. Elyamn has nothing to disclose. E. E. Evans is an employee and stockholder, Vaccinex, Inc. P. Fitzgerald‐Bocarsly has nothing to disclose. K. E. Foley has nothing to disclose. A. S. Harms has nothing to disclose. M. T. Henea has nothing to disclose. S. Hong has acted as a paid consultant to Eisai Ltd, Novo Nordisk, and Alnylam; receives research funding from AstraZeneca and Eisai Ltd; and has a collaborative project with Ionis Ltd. Y. A. Huang has nothing to disclose. S. Jackvony has nothing to disclose. L. Lai has nothing to disclose. Y. Le Guen has nothing to disclose. C. Lemere has nothing to disclose. S. A. Liddelow maintains a financial interest in AstronauTx Ltd and Synapticure. A. Martín‐Peña has nothing to disclose. A. G. Orr has nothing to disclose. F. J. Quintana has nothing to disclose. G. D. Ramey has nothing to disclose. J. E. Rexach has nothing to disclose. S. J. S. Rizzo has served as a consultant for Hager Biosciences, Genprex, Inc., and Sage Therapeutics, and holds shares in Momentum Biosciences. C. Sexton is an employee of the Alzheimer's Association. A. S. Tang has nothing to disclose. J. G. Torrelas has nothing to disclose. A. P. Tsai has nothing to disclose. L. Van Olst has nothing to disclose. K. A. Walker has nothing to disclose. W. Wharton has nothing to disclose. M. G. Tansey is a co‐inventor on the DN‐TNF (XPro1595) patent and a consultant to INmune Bio. which is developing the biologic for neurological indications. M. G. Tansey is a member of the MSAG at the Alzheimer's Association. D. M. Wilcock is Editor‐in‐Chief, of Alzheimer's & Dementia. Paid services for Novo Nordisk. Travel support from ADPD and Alzheimer's Association. Author disclosures are available in the supporting information.

## Supporting information



Supporting Information
